# Systemic sclerosis-associated interstitial lung disease: How to manage in 2024?

**DOI:** 10.2478/rir-2024-0022

**Published:** 2024-10-21

**Authors:** Rocio Bautista-Sanchez, Dinesh Khanna

**Affiliations:** Division of Rheumatology, University of Michigan, Ann Arbor, MI, Michigan USA; Scleroderma Program, University of Michigan, Ann Arbor, MI, Michigan USA

**Keywords:** interstitial lung disease, systemic sclerosis, management, treatment, antifibrotics, immunosuppression

## Abstract

Systemic sclerosis (SSc) or scleroderma is an autoimmune disease characterized by immune dysregulation which leads to progressive fibrosis of the skin and internal organs. Interstitial lung disease (ILD) is present in approximately 65% of patients with SSc and it accounts for approximately 40% of all SSc deaths. Risk factors associated with the development of systemic sclerosis related interstitial lung disease (SSc-ILD) include male sex, African heritage, high modified Rodnan skin score (mRSS), presence of anti-Scl-70/Topoisomerase I antibodies, and nucleolar pattern on antinuclear antibody (ANA). The primary tool to diagnose ILD in patients with SSc is high-resolution computed tomography (HRCT). Full pulmonary function tests (PFTs) with diffusing capacity of the lungs for carbon monoxide (DLco) and ambulatory desaturation testing should be obtained following the diagnosis of SSc-ILD for disease monitoring.

The purpose of this review is to provide an updated guide for the management of SSc-ILD. Our proposed first line treatment for SSc-ILD is immunosuppressive therapy such as mycophenolate mofetil, tocilizumab, and rituximab which are discussed in depth, and we present the evidence-based data that has justified the use of these pharmacotherapies. Other immunosuppressive treatments are also reviewed, and we discuss the role of antifibrotic therapy. Finally, we dive into other avenues of treatments such as chimeric antigen receptor (CAR)-T cell therapy and hematopoietic stem cell transplant.

## Background

Systemic sclerosis (SSc) or scleroderma is an autoimmune disease characterized by immune dysregulation which leads to progressive fibrosis of the skin, internal organs, and vasculopathy.^[1]^ Pathophysiologic mechanisms have been extensively described which include microvascular injury and endothelial dysfunction, dysregulation of the innate and adaptive immunity, and fibrosis of tissues.^[2]^ Interstitial lung disease (ILD) is present in 50%–80% of patients with SSc and, although it is only clinically significant in 25%-30% of these patients, it accounts for approximately 40% of all SSc deaths.^[1,2]^ The increased awareness of these alarming statistics has encouraged the medical and scientific community to further the efforts of early detection, prompt treatment, and diligent monitoring of individuals with systemic sclerosis related interstitial lung disease (SSc-ILD).

## Screening and Risk Factors

Screening has been recognized as an imperative part of successful management of SSc-ILD. In recent years multiple expert consensuses in the United States and Europe agree that all patients with SSc should be screened for ILD with chest auscultation, high-resolution computed tomography (HRCT), full pulmonary function tests (PTFs), spirometry with DLco, and autoantibody testing at the time of diagnosis.^[3,4]^ Even with addition of diffusing capacity of the lungs for carbon monoxide (DLco), PFTs alone are inadequate to screen for SSc-ILD due to a sensitivity of 85%, meaning approximately 15% of patients with disease could have normal PFTs. Therefore, the use of HRCT on initial screening is highly recommended.^[5]^

Risk factors associated with the development of SSc-ILD include male sex, African heritage, high modified Rodnan skin score (mRSS), presence of anti-Scl-70/Topoisomerase I antibodies, and nucleolar pattern on antinuclear antibody (ANA).^[3]^ ILD has been consistently considered a poor prognostic factor in patients with SSc, especially patients with diffuse cutaneous systemic sclerosis (dcSSc) who are known to be at a higher risk within the first five years of symptom development.^[6]^ A reduced forced vital capacity (FVC) within four years of symptom onset has served as an important predictor of the subsequent development of severe lung disease in patients with scleroderma.^[6,7]^ Although majority of patients develop ILD in the first 3–5 years, recent data from a multicenter observational cohort and a single center study highlight that a proportion of patients may develop ILD during next several years after onset of SSc, requiring careful screening and follow up.^[8]^

## Diagnosis and Disease Monitoring

The primary tool to diagnose ILD in patients with SSc is HRCT, with DLco and FVC being supporting tools.[4,9,10] In 2023 the American College of Rheumatology (ACR) released a clinical guideline summary to approach ILD screening and monitoring in individuals with systemic autoimmune rheumatic diseases. The ACR emphasized repeating full PFTs with DLco every 3–6 months during the first year following the diagnosis of SSc-ILD, encouraged the use of ambulatory desaturation testing every 3–12 months, and repeat HRCT as deemed clinically necessary.^[9]^ HRCT is generally not done on yearly basis due to radiation risks.

In patients with an established diagnosis of SSc-ILD it is important to routinely screen for pulmonary arterial hypertension particularly when the degree of dyspnea is not explained by ILD progression^[1,3,6]^ There is pertinent prospective data that unveils an increase mortality risk among SSc-ILD patients with > 20% of parenchymal involvement on HRCT or with FVC < 70%, therefore diagnosing and stratifying patients appropriately is a critical part of the clinical practice.^[11]^

## Which Patients with SSc-ILD Require Treatment?

In recent years there has been an ongoing debate concerning which patients with SSc-ILD meet criteria for treatment initiation. These conversations are rooted in the knowledge that some asymptomatic individuals with small extent of parenchymal disease or lower risk antibody profile (anticentromere antibody positive) might have less risk of developing progressive or clinically significant disease, contrasted with the risks that ILD therapies confer due their immunosuppressive properties. Patients with SSc-ILD have been classified into subclinical ILD or clinical ILD based on initial presentation.

Subclinical ILD is defined by the presence of ILD with less than < 10% extent on HRCT and normal initial PFT (including FVC and DLco) without clinically relevant decline on serial measurements and no ILD-related symptoms (dyspnea, cough, *etc*.).^[12]^ For this group of patients, close monitoring may be acceptable unless the patient has a high risk disease profile such as anti-topoisomerase 1 antibody, elevated acute phase reactants, or rapidly progressive skin disease (“inflammatory subset”).^[3,4,12]^ These recommendations are focused on ILD and a patient with skin or other internal organ progression may be a candidate for immunosuppressive treatment.

The 2023 expert consensus on SSc-ILD management recommended immediate treatment initiation with immunosup-pression therapy in patients with moderate-to-severe ILD on HRCT (or > 20% lung involvement), FVC and/or DLco below the normal lower limit, early rapidly progressive dcSSc (even if only mild abnormalities are present on HRCT and/or PFTs), hypoxemia at rest, or desaturations on exercise. Treatment was also recommended in patients with abnormal or progressive findings on HRCT, FVC < 80%, or FVC > 80% if accompanied by ILD in a high-risk patient.^[3]^

## Treatment of Systemic Sclerosis-Associated Interstitial Lung Disease

Preventive health measures are fundamental in patients with SSc-ILD as there is an increased risk of infection due to altered anatomy and physiology. There is also a risk of disease progression with recurrent lung injury. We recommend all patients with SSc-ILD to routinely receive pneumococcal, annual influenza, and corona virus disease 2019 (COVID-19) immunizations. The newly available respiratory syncytial virus (RSV) vaccine should also be considered in this high-risk population. Gastroesophageal reflux disease (GERD) is a common manifestation of SSc and if uncontrolled increases the risk of aspiration leading to chemical pneumonitis or aspiration pneumonia furthering lung injury, therefore, we recommend diligent treatment of GERD in patients with SSc-ILD.

### Preferred First Line Treatment

**Figure 1:**
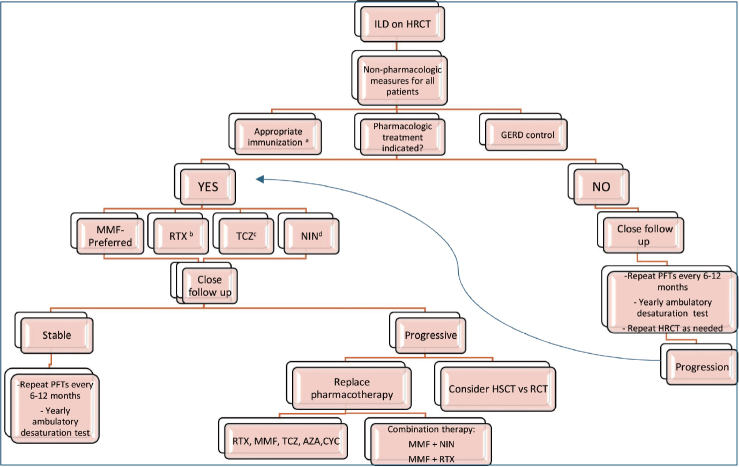
Suggested treatment algorithm for systemic sclerosis-associated interstitial lung disease. A. COVID-19, influenza, pneumococcal, and RSV vaccine are recommended. B. Requires less frequent administration which can improve medication adherence; can be considered if inflammatory arthritis is present. C. Consider in patients with elevated inflammatory markers and inflammatory arthritis. D. Consider as primary therapy if predominantly fibrotic pattern on HRCT and quiescent extra pulmonary disease, NIN will not treat extrapulmonary disease. HRCT, high-resolution chest computerized tomography; GERD, gastroesophageal reflux disease; MMF, Mycophenolate mofetil; RTX, Rituximab; TCZ, tocilizumab; NIN, Nintedanib; PFT, pulmonary function testing; HSCT, hematopoietic stem cell transplant; RCT, randomized clinical trial; AZA, azathioprine; CYC, cyclophosphamide.

Our first line treatment is immunosuppressive therapy for SSc-ILD with rare exceptions (Figure 1).

#### Mycophenolate Mofetil

Mycophenolic acid, the active metabolite of mycophenolate mofetil (MMF) and mycophenolate sodium (MS), is an inhibitor of inosine monophosphate dehydrogenase that reversibly impairs T and B cell proliferation without causing myelotoxicity. Also, clinical and in vitro data suggest that it can affect fibroblast biology, thus producing antifibrotic and immunomodulatory effects.^[13,14]^

The Scleroderma-Lung Study (SLS) II was a multicenter, randomized, double-blind clinical trial that compared MMF at a target dose of 1500 mg twice daily for 24 months to oral cyclophosphamide (CYC) at a target dose of 2 mg/kg/day for 12 months followed by placebo for 12 months. MMF was found to be non-inferior to CYC with similar improvement in forced vital capacity percentage (FVC%), mRSS, self-reported dyspnea, and the quantitative extent of ILD (QILD) in the whole lung.^[15]^ MMF appeared to be better tolerated with a lower incidence of treatment withdrawal, leukopenia, and thrombocytopenia. MMF was recommended as the first line treatment for SSc-ILD by the 2023 ACR guidelines and the recently published American Thoracic Society (ATS) guidelines for SSc-ILD management given the reduction in disease progression, improvement in quality-of-life measures, and minimal adverse events.^[9,16]^

MS is known to have a similar mechanism of action to MMF and given its delayed release properties provides less gastrointestinal side effects. Various observational studies have evaluated MS for SSc-ILD with mixed results and a lack of power has been the main limitation.^[17,18]^ Lastly, it is important to indicate that participants of the SLS-II had abnormal FVC with dyspnea on exertion, therefore, future research is required to evaluate MMF benefits in patients with early ILD, including the “inflammatory subset”.

#### Tocilizumab

Tocilizumab (TCZ) is an anti-interleukin 6 (IL6) receptor monoclonal antibody. Two major studies solidified the use of TCZ in SSc-ILD. Interestingly, both studies failed to meet their primary end point which was a reduction in mRSS.^[19]^

The faSScinate trial was a phase 2 trial in patients with early dcSSc with less than 5 years since the onset of the first non-Raynaud’s phenomenon manifestation of SSc. 87 patients were randomized to weekly 162 mg of subcutaneous TCZ or placebo for 48 weeks. Primary endpoint was improvement in mRSS at 24 weeks which was not met; however, secondary analysis revealed that TCZ reduced the rate of FVC% decline among the study arm (-2.6% [-5.2 to -0.1]) compared to the placebo arm (-6.3% [-8.9 to -3.8]) at 48 weeks.^[20]^

The focuSSced phase 3 trial studied a similar population with early dcSSc and included 210 patients, 104 were randomized to receive weekly TCZ 162 mg injection, and 106 were randomized to placebo. Primary endpoint was change from baseline in mRSS at week 48 which was not met, although participants treated with TCZ had a numerically greater reduction in skin sclerosis. Change in FVC% predicted at week 48 and time to treatment failure were secondary endpoints. The baseline FVC% predicted in the TCZ arm was 80 (±14) and 84 (±15) in the placebo arm. Among the participants with SSc-ILD 67% of patients in the placebo group exhibited significant rate of decline in FVC% compared to the TCZ group at 48 weeks (-6.3% *vs*. -0.1%) with a total difference of 6.2% (nominal *P* < 0.0001). Importantly, there was also significant improvement on HRCT involvement in the TCZ arm compared to the placebo arm.^[21,22]^ In a post-hoc analysis, the placebo group had a large decline, irrespective of the degree of ILD at baseline.

An open label extension of the focuSSced trial ran from weeks 48 to 96 in which all participants received TCZ treatment. Two groups were identified the continuous-TCZ arm (*n* = 82) *vs*. the placebo-TCZ arm (*n* = 85). Participants in the continuous-TCZ treatment had preserved lung function, and the placebo-TCZ group experienced improvement in lung function following addition of TCZ by week 96.^[23]^ The trial showed preservation of lung function in SSc-ILD, and the open label highlighted the potential reversibility in the lung function decline once the patients on placebo were started on TCZ. TCZ can be considered as first line therapy in patients with early SSc with progressive skin disease and either with elevated acute phase reactants or anti-Scl-70 ab as 68% had presence of this antibody.

#### Rituximab

B cells have been implicated in the pathogenesis of SSc. Patients with scleroderma exhibit blood abnormalities such as an expanded naïve B cell population and diminished but hyperreactive memory B cells with CD19 overexpression.^[24]^ Rituximab (RTX) is a chimeric monoclonal antibody that targets the CD20 receptor of B cells and eradicates them, therefore, it has become a therapy of interest for SSc-ILD.

An open label, randomized, controlled trial evaluated CYC versus RTX in 60 treatment naïve, anti-Scl-70 positive, early dcSSc-ILD patients. Patients were randomized to receive monthly CYC (500 mg/m^2^) for 6 months or RTX (1000 mg) at 0, 15 days. The primary endpoint was predicted FVC% improvement. Absolute FVC change in liters, mRSS, and 6-minute walk test were secondary endpoints. At 6 months, the RTX group exhibited improved FVC% from 61.30 to 67.52 while the CYC group had declined from 59.25 to 58.06 (*P* = 0.003). The RTX arm also experienced a statistically significant improvement of the mRSS, 6-min walking test, and a better side effect profile over the CYC arm.^[25]^

The Rituximab versus cyclophosphamide for the treatment of connective tissue disease-associated interstitial lung disease (CTD-ILD) or RECITAL was a randomized, double-blind, double dummy trial which included patients with severe or progressive ILD and a confirmed diagnosis of SSc, idiopathic inflammatory myositis (polymyositis or dermatomyositis), or mixed connective tissue disease. A total of 101 patients were allocated to receive CYC (600 mg/m^2^ every 4 weeks for 6 months) or RTX (1000 mg at weeks 0 and 2). The hypothesis was RTX would be superior to CYC. The primary outcome was the change in FVC measured in mL by week 24.48 (96%) participants in the CYC group and 49 (96%) patients in the RTX group received at least one dose of treatment and were included in the modified intention-to-treat population for primary outcome analysis. The CYC arm had a mean FVC gain of 99 mL and the RTX group 97 mL. There were no differences in groups at week 24 (*P* = 0.49) or week 48 (*P* = 0.345). Similar improvements were also seen in DLco, 6-minute walk test, global quality of life in both treatment groups. The RTX group required 12.3% less corticosteroid use and experienced less adverse events. Finally, the effects of treatment were consistent across the three different CTD subgroups.^[26]^

The aforementioned studies have consolidated the use of RTX for SSc-ILD at the time of initial diagnosis and in patients with severe and progressive disease. In our practice, RTX can be considered first line therapy for those with inflammatory arthritis or those who prefer every 6-month infusion therapy.

### Additional Therapies

Other immunosuppressive pharmacotherapies to consider include cyclophosphamide and azathioprine or the antifibrotic agent Nintedanib.

#### Cyclophosphamide

CYC was the preferred agent to treat patients with SSc-ILD. These recommendations were substantiated by the Fibrosing Alveolitis in Scleroderma Trial (FAST) and SLS-I.^[6]^ The FAST trial ran for 12 months and evaluated monthly intravenous CYC (600 mg/m^2^) plus prednisone 20 mg every 48 h for 6 months, followed by azathioprine (2.5 mg/kg per day) *vs*. placebo. There was a trend towards improvement of FVC in the active treatment group, but DLco, total lung capacity (TLC), disease extent on HRCT, and dyspnea scores were not significantly different between groups.^[27]^

The SLS-I assessed oral cyclophosphamide (2 mg/kg per day) for 12 months or placebo. The treatment group exhibited statistically significant improvement of FVC, TLC, dyspnea scores, and functional ability.^[28]^ Despite the apparent favorable outcomes of the SLS I, there were two reasonable concerns: (1) The side effect profile of oral CYC concerning hematologic toxicity, hematuria, increased risk of infections, and infertility; (2) The lack of sustained benefit following CYC discontinuation.^[29]^ The toxicity of CYC and availability of equally effective therapies has made CYC a less preferred therapy in our practice. We acknowledge that in certain countries, the cost of CYC is less and therefore utilized as first line treatment.

#### Nintedanib

Nintedanib (NIN) is an oral tyrosine kinase inhibitor known to inhibit processes and pathways involved in fibrogenesis.^[16]^ Nintedanib is known to have predominantly antifibrotic properties. The SENSCIS trial was a phase 3, randomized, controlled study in which 576 participants with SSc-ILD and > 10% of lung fibrosis of HRCT were enrolled to receive NIN 150 mg twice per day (*n* = 288) or placebo (*n* = 288) over a 52-week period. Background therapy with MMF was allowed and approximately 50% of patients in both arms received it. The primary endpoint was rate of decline in FVC at week 52. The average FVC% predicted was 72.4 in the NIN arm and 72.7 in the placebo arm. At 52-week the NIN arm demonstrated a reduction in the annual rate of decline of FVC (-52.4 mL) compared to the placebo arm (-93.3 mL). There was a total FVC% predicted difference of 1.2% between groups favoring NIN (95% CI: 0.1–2.2). Overall, the NIN group experienced more nausea, vomiting, diarrhea, and weight loss leading to treatment discontinuation.^[30]^

The post hoc analysis of the SENSCIS trial found significant data on the minimal clinically important difference (MCID). 34.5% of subjects in the NIN group versus 43.8% of subjects in the placebo had an absolute decrease in FVC of ≥3.3% predicted at week 52 (the proposed MCID for worsening of FVC), while 23.0% *versus* 14.9% had an absolute increase in FVC of ≥3.0% predicted at week 52 (the proposed MCID for improvement in FVC).^[16,31]^

Nintedanib has been conditionally recommended as therapy for SSc-ILD by the ATS and ACR guidelines. The main limitations include poor tolerability generally due to gastrointestinal side effects and lack of effect on the extra pulmonary aspects of the SSc. The results of the SENSIS trial favored the use of this agent, and we incorporate NIN as add on therapy to immunosuppressive treatment. It may be reasonable first line agent in patients with a predominantly fibrotic pattern SSc-ILD (such as usual interstitial pneumonia (UIP) pattern) and quiescent extra pulmonary disease, or patients who get recurrent infections on immunosuppressive therapy. Although there is lack of data, upfront combination therapy may be considered with MMF or other immunosuppressive therapies in rapidly progressive SSc-ILD.

#### Azathioprine

Azathioprine (AZA) is a purine analogue that when converted into its active metabolites inhibits purine synthesis and incorporates into replicating DNA halting the process. AZA has been considered an effective drug for maintenance therapy in patients with SSc-ILD following the SLS-I.^[28]^ An Australian scleroderma cohort study evaluated patients with SSc-ILD and compared AZA *vs*. MMF treatment assessing the rate of decline in lung function before and after therapy initiation. There were no differences between groups, although patients treated with AZA experienced more gastrointestinal distress, cytopenias, hepatotoxicity, and rashes.^[32]^ Although there is limited data to suggest the use of AZA as a preferred agent, its known favorable safety profile in pregnancy makes it one to consider.

## Progressive SSc-ILD and Management

### What Is Progressive SSc-ILD?

Many definitions of progressive ILD have been included in different trials and cohorts. Progressive ILD has been defined as a decrease in FVC ≥10%, or FVC decline of ≥ 5% with a drop in DLco of 10%–15% (corrected for hemoglobin) after one year of a designated ILD therapy.^[6,11,12]^ Other definitions include a combination of decline in PFTs, HRCT with progressive disease, and increasing ILD specific symptoms.^[33]^ These parameters have been used to stratify patients and predict survival.

### What Are the Preferred Pharmacologic Agents to Treat Progressive SSc-ILD?

MMF, CYC, TCZ, RTX, and NIN have shown to be effective for progressive SSc-ILD. CYC has faced head-to-head trials against multiple other therapies such as MMF (in the SLS-II) and RTX (in the RECITAL trial), and although its efficacy has been well stablished, especially in progressive SSc-ILD, the side effect profile tends to be less favorable. On the other hand, RTX and MMF have proven to be non-inferior and effective for patients with progressive disease. Additionally, patients with dcSSc are more likely to develop progressive ILD in the first 5 years of disease onset, therefore, TCZ can be considered in patients with elevated inflammatory markers.

### Is There Evidence for Pharmacologic Combination Therapy?

#### RTX Plus MMF

Combination therapy for progressive SSc-ILD is an interesting area of research with promising results. The recently published EVER-ILD trial was a multicenter, randomized, double-blind, placebo-controlled study that evaluated RTX + MMF versus MMF alone in patients with CTD-ILD or idiopathic interstitial pneumonia, and non-specific interstitial pneumonia (NSIP) on histopathology or NSIP-like pattern on HRCT. A total of 126 patients received either RTX (1000 mg) on day 1 and day 15 + MMF (2 g daily) for 6 months OR placebo + MMF for 6 months. A total of 43 patients (35%) had CTD, of which 23 (53%) had SSc. Primary end point was change in absolute FVC % from baseline to 6 months. At 6 months the RTX + MMF group had approximately 3.6% increase of absolute predicted FVC when compared to the placebo + MMF arm (95% CI: 0.41–6.80; *P* = 0.0273). Progression free survival was also greater in the RTX+MMF group (crude hazard ratio 0.47, 95% CI: 0.23–0.96; *P* = 0.03). There were no significant differences observed in 6-min walk distance, DLco, HRCT ILD extent, or adverse event rates.^[34]^ Nonetheless, the control arm did not receive a maximum dose of MMF (3 g daily) and perhaps this contributed to the perceived amplified benefit of combination therapy with RTX + MMF.

#### Nintedanib plus MMF

A SENSCIS trial post Hoc analysis found significant differences between the subgroup of patients who received NIN + MMF versus the placebo group. The combined therapy subgroup had 2.5% lower annual rate of decline in predicted FVC% compared to the placebo subgroup. Interestingly, there were no significant differences in the NIN + MMF subgroup when compared to the MMF or NIN alone groups.^[31]^ The main limitations to the use of combination therapy are poor tolerability (which may lead to treatment withdrawal) and decreased quality of life. Combination therapy was associated with a sevenfold higher risk of decreased appetite, more than 2.5-fold higher risk of diarrhea, and about threefold higher risk of nausea, vomiting, and/or fatigue compared with placebo.^[16]^ Nonetheless, given its clear benefit, it is conditionally recommended in patients with SSc-ILD.

#### Pirfenidone plus MMF

Pirfenidone (PFD) is an antifibrotic therapy although its mechanism of action is not fully known. The Scleroderma Lung Study III (SLS-III) compared PFD + MMF therapy to MMF alone in patients with SSc-ILD. While the official study results have not been yet published, an abstract is available for review. The study was aborted given recruitment difficulties during the COVID-19 pandemic. A total of 51 patients were recruited (the target was 150), therefore the study was underpowered. The primary endpoint was the change from baseline in the mean FVC% over the course of the 18-month treatment period. 27 patients were enrolled in the MMF + PFD arm and 24 to MMF+ placebo. Similar rates of improvement in FVC% were seen in both arms at 18 months (2.24% MMF + placebo *vs*. 2.09% MMF + PFD; *P* = 0.93).^[35]^ The evidence for the use of PFD at present is low, therefore, the ATS guidelines for the management of SSc-ILD have cited insufficient evidence to make a recommendation.

## What Pharmacologic Therapies Should Be Avoided in Patients with SSc-ILD?

Glucocorticoid (GC) treatment has been strongly recommended against for the management of SSc-ILD as initial treatment strategy, and its long-term use is strongly discouraged in progressive stages of the disease given the increased risk for scleroderma renal crisis.^[9]^ In addition, GC has not shown to be effective in early SSc-ILD since it is predominantly a fibrotic (rather than cellular) ILD.

## Hematopoietic Stem Cell Transplant

Three randomized controlled trials have shown favorable results concerning autologous hematopoietic stem cell transplant (AHSCT): ASSIST, ASTIS, and SCOT.

The ASSIST trial in 2011 enrolled 19 patients < 60 years old with dcSSc, mRSS > 14, and internal organ involvement (lung, renal, or cardiac) who received non-myeloablative therapy (2 g/m^2^ CYC) plus AHSCT or intravenous cyclophosphamide (1 g/m^2^) every month for 6 months. At 1-year FVC had increased by 15% in AHSCT group and declined by 9% in CYC arm (*P* = 0·006), mRSS improved in the transplant group and worsened in the mRSS group. The transplant arm also had greater improvement in the volume of affected lung in HRCT.^[36]^ The ASTIS trial (2014) had a similar study design and included 156 patients who were randomly assigned to receive AHSCT (*n* = 79) or cyclophosphamide (*n* = 77), patients had median follow-up of 5.8 years. Event-free survival hazard ratio (HR) at 1 year was 0·52 (*P* = 0·04) and at 2 years was 0·35 ( *P* = 0·006) in the AHSCT arm. Although there were statistically significant improvements in TLC and FVC, most patients had mild ILD with an average FVC > 80% and only moderately decreased DLco.^[37]^

The SCOT trial published in 2018 enrolled 75 patients with severe SSc who were randomized to receive myeloablative regimen (CYC 120 mg/kg total dose PLUS total body irradiation PLUS equine antithymocyte globulin) followed by AHSCT or CYC (500 mg/m^2^ once, followed by 11 monthly infusions of 750 mg/m^2^). The primary endpoint was improvement in the global rank composite score (GRCS), an analytic tool which includes multiple disease features, including death and event-free survival, FVC, the Disability Index of the Health Assessment Questionnaire (HAQ-DI) score, and the mRSS. After 54 months 67% of patients treated with HSCT reached the primary endpoint, the improvement of GRCS when compared with 33% in the CYC arm.^[38,39]^

## Chimeric Antigen Receptor-T Cell Therapy

Chimeric antigen receptor (CAR)-T cell therapy has been a groundbreaking treatment used for multiple oncologic indications in recent years. CARs are engineered synthetic receptors that redirect T-cell lymphocytes to recognized and eliminate cells expressing a specific antigen. CAR binding to target antigens expressed on the cell surface is independent from the major histocompatibility complex (MHC) receptor resulting in vigorous T cell activation and powerful anti-tumor responses.^[40]^

Recently, third generation CD19-CAR-T cells were utilized in a Scl-70 ab positive patient with SSc-ILD and progressive pulmonary fibrosis despite treatment with CYC, followed by MMF + NIN therapy. Although the effects of CAR-T therapy were not perceived immediately, 5 months following CAR-T infusion there was improvement of PFTs, fibrosis scores on HRCT, mRSS, and symptomatology.^[41]^ Two other case reports and a recently published case series of CAR-T use in patients with autoimmune diseases and ILD have revealed promising data with longitudinal follow up of two years.^[42,43,44]^

Additional trials are needed to confirm the efficacy, safety, and durability of response of CAR-T in SSc.

## Conclusion

ILD remains one of the most challenging manifestations of SSc and is known to increase the morbidity and mortality drastically among patients. Recent scientific advances have yielded a deeper understanding of the different pathophysiologic mechanisms of this disease. Our goal is to equip physicians with an updated and thorough summary that approaches screening strategies, diagnostic tools, and evidence-based therapy options that will ensure successful triage and management of patients with SSc-ILD. The available pharmacotherapy provides options to treat inflammatory, fibrotic, or mixed phenotypes of ILD. Most importantly, emerging treatment strategies such as HSCT and CAR-T cell therapy may revolutionize the future of SSc-ILD.
